# Computational Modeling of Virally-encoded Ion Channel Structure

**DOI:** 10.21203/rs.3.rs-2182743/v1

**Published:** 2022-10-19

**Authors:** Alexander Weissman, Jeremy Bennett, Nicole Smith, Carly Burdorf, Emma Johnston, Beth Malachowsky, Lori Banks

**Affiliations:** Bates College; Bates College; Bates College; Bates College; Bates College; Bates College; Bates College

**Keywords:** rotavirus, viroporin, structure prediction, AlphaFold, trRosetta

## Abstract

Viroporins are ion channels encoded within a virus’s genome, that facilitate a range of devastating infectious diseases such as COVID-19, HIV, and rotavirus. The non-structural protein 4 (NSP4) from rotavirus includes a viroporin domain that disrupts cellular Ca2+ homeostasis, initiating viral replication, and leading to life-threatening vomiting and diarrhea. Though the structure of soluble segments of NSP4 has been determined, membrane-associated regions, including the viroporin domain, remain elusive when utilizing well-established available experimental methods such as x-ray crystallography. However, two recently published protein folding algorithms, AlphaFold2 and trRosetta, demonstrated a high degree of accuracy, when determining the structure of membrane proteins from their primary amino acid sequences, though their training datasets are known to exclude proteins from viral systems. We tested the ability of these non-viral algorithms to predict functional molecular structures of the full-length NSP4 from SA11 rotavirus. We also compared the accuracy of these structures to predictions of other experimental structures of eukaryotic proteins from the Protein Data Banks (PDB), and show that the algorithms predict models more similar to corresponding experimental data than what we saw for the viroporin structure. Our data suggest that while AlphaFold2 and trRosetta each produced distinct NSP4 models, constructs based on either model showed viroporin activity when expressed in E. coli, consistent with that seen from other historical NSP4 sequences.

## Introduction

Rotavirus (RV) is a vaccine-preventable infectious agent, chiefly responsible for most viral gastroenteritis in young children across the world. In fact, prior to the introduction of the first rotavirus vaccine in 1998, it was estimated that 95% of children worldwide were infected with RV gastroenteritis by the age of five, and caused roughly 450,000 under-five deaths annually [1–4]. The introduction of the RV vaccine has resulted in a drop of annual death count to approximately 200,000. Unfortunately, most of these preventable annual deaths originate from low-income countries [3, 5]. These deaths are typically also met with numerous comorbid conditions, including malnutrition, lack of available potable water, and limited access to health care [3]. These comorbidities translate to decreased vaccine efficacy, from 85–100% in middle- and high-income countries, down to 48–61% in low-income countries [1, 6]. Uncovering the mechanisms of RV pathogenesis will provide insight into developing therapies to more effectively counter viral gastroenteritis.

Rotaviruses are non-enveloped, icosahedral, double-stranded RNA viruses in the Sedoreovirinae subfamily of the Reoviridae family. Its three capsids surround a genome of 10–12 segments of dsRNA (varying among the different genera) encoding six structural and six non-structural viral proteins [1, 6]. RV structural proteins allow for viral-host specificity, cell entry, and enzymatic activity, whereas the non-structural proteins are responsible for genome replication, and the deactivation of the host’s innate immune response [3]. RV non-structural protein 4 (NSP4) plays a vital role in RV replication, morphogenesis, and pathogenesis. Therefore, NSP4 has proved to be a fascinating target for antiviral drug design.

NSP4 is a transmembrane glycoprotein which is initially synthesized in the endoplasmic reticulum (ER) [7]. In the ER, NSP4 plays a key role in virus maturation. When NSP4 is active, it releases Ca^2+^ from the ER, elevating cytosolic Ca^2+^ in eukaryotic cells [8]. This in turn activates Ca^2+^ activated Cl- channels, resulting in the secretion of Cl- in the lumen of mammals, the primary pathological mechanism which causes RV gastroenteritis. NSP4 is a multifunctional protein consisting of a viroporin domain (VPD, residues 47–90) and a coiled-coil domain (CCD, residues 95–137), motifs that are commonly associated with other virus-encoded ion channel proteins (viroporins) [7, 8]. NSP4 has also been shown to secrete an enterotoxin cleavage product (Enterotoxin, residues 112–175) from infected cells [9]. The exact demarcation of each of these domains has not been clearly defined. Structurally, viroporins typically consist of 60–120 amino acids. Although viroporins target a wide range of intracellular components, this class of proteins tends to share secondary structural motifs, such as amphipathic alpha-helices and clusters of basic residues [10]. The electrostatic properties of these common motifs aid in viroporin insertion into the host cell membrane, and ultimate conductance of cations across a membrane. Based on studies of SA11 NSP4 from Hyser et al., these common motifs are apparent within the VPD (amino acids 47–90), where an amphipathic helix, and a helix exhibiting five lysine residues, have been found to be critical for NSP4 transmembrane insertion and Ca^2+^ ion conductance [7, 9]. In addition to the determination of secondary structures within NSP4, crystallographic studies have shown that the CCD of NSP4 has two oligomeric states, a Ca^2+^ bound tetrameric conformation and an ion-free pentameric conformation. The oligomeric state of NSP4 appears to be affected by changes in pH [7]. Apart from these crystallographic studies focusing on the soluble domain of NSP4, the structure of the membrane-associated VPD is unknown.

In vitro methods of deducing protein structure, which include cryo-EM, NMR, and x-ray crystallography, have several drawbacks. For one, some existing in vitro methods struggle to produce physiologically accurate structures, especially when there are no known homologues [11]. Additionally, the cost to generate structural models from these methods is quite high, which can limit completion of these studies. Furthermore, the time commitment can be many months to years, a drawback that has been exacerbated by supply chain shortages during the COVID-19 pandemic. Given the critical role of this viroporin in RV pathogenesis, it is vital to determine the full-length structures of the NSP4 multimers, in order to fully understand the various functions of each of its domains. However, previous attempts to do so have been bottlenecked by the protein-folding problem: the inability to determine the molecular structure of a protein, due to experimental limitations on producing a sufficient quantity of purified protein.

First emerging in 1960, the protein folding problem has proven to be among the most elusive mysteries in modern biochemistry. The discovery of the first atomic-resolution protein structures gave rise to the question of how a protein’s primary structure dictates its secondary, tertiary, and quaternary structure(s) [12]. Hypothesizing that the development of accurate in silico-based protein structure modeling methods could allow for modeling from genome sequences, potentially replacing in vitro methods, John Moult founded the Critical Assessment of Techniques for Protein Structure Prediction (CASP) in 1994 [13]. CASP is a biennial meeting within the international community of computational biology, to test the effectiveness of structure-prediction algorithms, and increase the number of newly available protein structure models [12].

AlphaFold2, released during CASP14 in 2020, was the first algorithm of its kind to regularly predict protein structures with near experimental accuracy, even when there was no known homologous structure [11]. Interestingly, for many of the predictions where AlphaFold2 disagreed with the experimentally determined structures, the margin of error from both models was so small that it was not entirely clear which was closer to the true structure. Although AlphaFold2 had the highest overall accuracy, it still showed difficulty in accurately predicting protein complexes. This was addressed in October 2021 with the release of AlphaFold-Multimer, a model trained specifically for multimeric inputs. It significantly increased the accuracy of predicted multimeric interfaces over single-chain AlphaFold2, while maintaining high intra-chain accuracy [14]. For homomeric interfaces, AlphaFold-Multimer successfully predicted the structure in 69% of cases. The marked increase in accuracy was made possible through this new use of deep learning algorithms of neural networks.

Neural networks consist of thousands (or even millions) of densely interconnected processing nodes, that are typically organized into layers in which data flows in one direction [15]. Deep learning refers to a subset of neural networks with at least three layers of nodes, consisting of an input layer, one or more succeeding layers, and a final output layer. An individual node is typically connected to several nodes in both the layers beneath and above it, receiving data from one end, and sending data through to the other [15]. Nodes function by assigning numbers called weights, to each incoming connection. When a node receives data from an incoming connection, this value is multiplied by its associated weight. If the resulting number exceeds the threshold value, the node “fires” and sends this value to its outgoing nodes. A fully trained neural network consists of properly interacting layers of nodes which can learn to recognize patterns within a training dataset. When neural networks are being trained, all of the weights and thresholds are set to random values. The output is then checked for errors against the training dataset. Data from the errors are used to adjust weights and thresholds over subsequent generations, until the neural network is able to consistently yield accurate outputs [15]. With each generation, weights and thresholds associated with proper function emerge, making the algorithm more accurate.

For protein folding neural networks, the training dataset consists of known protein structures from the Protein Data Bank (PDB). AlphaFold is unique from other structure prediction algorithms as it uses a secondary training dataset modeled after an approach called “noisy student self-distillation” [11]. The noisy student approach is a type of semi-supervised learning, allowing for the training of deep learning algorithms, with the use of labeled and unlabeled data, in three main steps: 1) train a teacher model using labeled data, 2) use the teacher model to assign “pseudo labels” to unlabeled data, and 3) train a student model using a combination of labeled and “pseudo labeled” data [16]. Using the student model as a teacher to train a new student can result in highly accurate recognition algorithms. The DeepMind team used a trained neural network to predict the structure of approximately 350,000 assorted sequences from the Uniclust30 database. A combination of their structure predictions, as well as PDB data, was used to train AlphaFold2.

Currently, the training datasets from both AlphaFold2 and trRosetta do not include viral proteins; as such, there are no analogous proteins for reference when building a structure. Viruses often encode polyproteins, which have exceedingly plastic structures that are often too unpredictable and taxing on computing resources to model. Because NSP4 is not cleaved off of a larger polyprotein, we believe this constraint is not applicable. Furthermore, both AlphaFold2 and trRosetta have recently been used, in addition to molecular dynamics (MD) simulations, to model the structure of severe acute respiratory syndrome coronavirus 2 (SARS-CoV-2) proteins, though these results have yet to be peer reviewed [17, 18]. Several SARS-CoV-2 proteins, including coronavirus NSP4, have not yet been modeled via in vitro methods, making these predictions the only available models.

Here, we present computational models of human, bacterial, and viral proteins, including SA11 rotavirus NSP4 monomer, tetramer, and pentamer, to weigh the strengths and weaknesses of AlphaFold2 and trRosetta, for determining these very divergent protein structures.

## Methods

### Computational Protein Modeling and Analysis

The primary amino acid sequence for SA11 rotavirus NSP4 was obtained from National Center for Biotechnology Information (NCBI) (accession number AF087678.1), and the sequences for both PETase (PDB:6EQF) and Sonic Hedgehog (PDB:3M1 N) proteins were obtained from the Research Collaboratory for Structural Bioinformatics Protein Data Bank (PDB) [19, 20]. These sequences were separately submitted to the Colab notebook version of AlphaFoldv2.1.0 and AlphaFold-Multimer in FASTA format, and transform-restrained Rosetta (trRosetta) to protein folding. trRosetta structure prediction was based on direct energy minimizations with a restrained Rosetta. These restraints included inter-residue distance and oriental distributions, which were predicted by a deep residual neural network. The outputs of these algorithms were downloaded as PDB files. Experimental structures for the NSP4 coiled-coil domain tetramer (4WB4) and pentamer (4WBA) were also included in the analysis [7]. Where mentioned, models were subsequently refined using Protein structure REFinement via Molecular Dynamics (PREFMD) [21]. Renderings of each model, and RMSD values generated, were created with PyMOL Molecular Graphics System, Version 2.0 (Schrödinger, LLC) for detailed analysis. Ramachandran plots were generated using PROCHECK [22, 23].

### Viroporin Assay

The viroporin activity of NSP4 truncation mutants was as previously described [6]. Plasmids encoding WT SA11 NSP4 residues 47–146 were transformed into BL21 (DE3) pLysS E. coli cells (Promega) via heat shock at 42°C for 30 seconds and then recovered at 37°C for 1 hour, prior to plating onto LB agar (Research Products International) containing 1% glucose, 50 mg/mL carbenicillin, and 37 mg/mL chloramphenicol. Single colonies were picked, and grown overnight at 37°C in LB broth containing 1% glucose, 50 mg/mL carbenicillin, and 37 mg/mL chloramphenicol, and shaken at 200 rpm overnight. The overnight culture was diluted into fresh media with the same composition, and grown until the optical density (OD) at 600 nm reached the range of 0.4–0.6. Next, Isopropyl ß-D-1-thiogalactopyranoside (IPTG) was added at a final concentration of 1 mM, and cultures were monitored for changes in 0D600 for 90 minutes. Measurements are reported as a fraction of the starting density of each culture.

## Results

**Figure 1:**
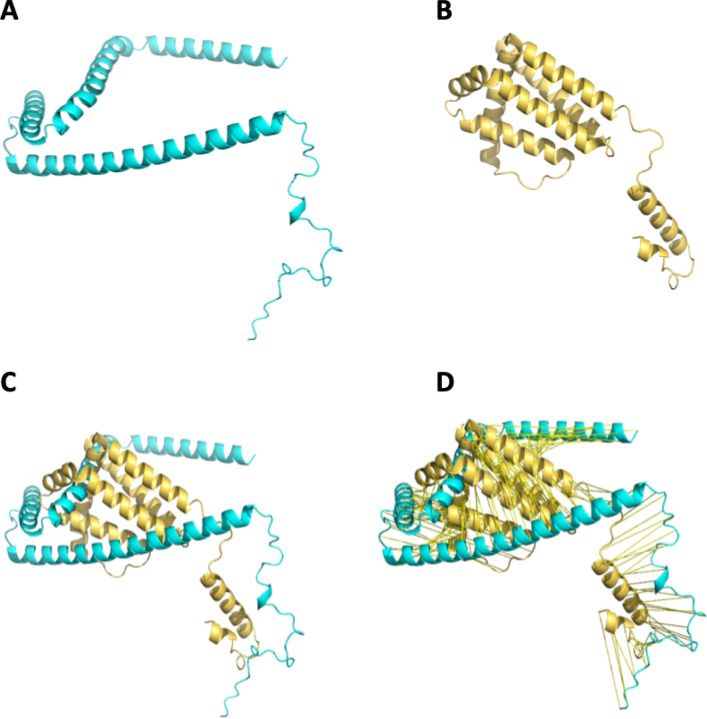
Computational models of NSP4 monomers show structural heterogeneity. Models of full-length WT rotavirus SA11 NSP4 monomers produced from **A.** AlphaFold2 (cyan), or **B.** trRosetta (gold), or the **C and D.** overlay of the two models (RMSD value of 23.70 Å) rendered using PyMOL. Homologous molecules are connected by yellow lines in **D.**

**Figure 2:**
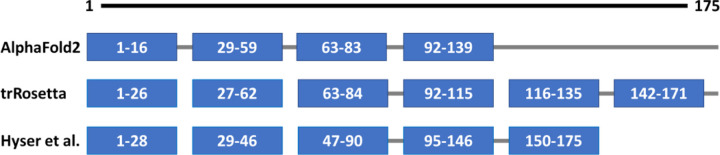
Computational models of NSP4 monomers exhibit differential segment assignment. Blue boxes represent predicted helices of each computational model spanning the amino acid numbers label within each (not drawn to scale) throughout the total 175 amino acids of the protein. Connecting loops or unordered regions between helices are shown as grey lines.

When comparing the AlphaFold2 model (Fig. 1A) and the trRosetta model (Fig. 1B), we found several structural differences. Aligning the two models resulted in a staggeringly high root-mean-square deviation (RMSD) value of 23.70 Å (Fig. 1C). RMSD is a measure of accuracy in which deviations between different models of a particular dataset are compared. An RMSD value of <3 Å is typical for homologous proteins. The trRosetta model predicted a greater number of helical sections, and coils, than the AlphaFold2 model. Furthermore, instead of predicting results consistent with the established limits of the VPD and CCD, this model instead split the entire structure into a series of approximately 26 amino acid long alpha helices (Fig. 2). Similarly, the AlphaFold2 model was not consistent with the established domain limits of the VPD assigned in part based on experimentally determined domains from viroporin-containing proteins from other viral systems [10]. However, the AlphaFold2 model’s prediction of the CCD was nearly the same as the established model from Hyser et al., which predicted the CCD to include residues 92–139, whereas the crystallographic data suggests the CCD to be at residues 95–137 [10]. Interestingly, additional molecular dynamics-based refinement of both models did not markedly change either the AlphaFold or trRosetta models as measured by alignment RMSDs between iterations of refinement (data not shown) [21, 24]. There were also no notable changes to the side-chain orientations of the calcium-binding site in the tetramer model.

**Figure 3:**
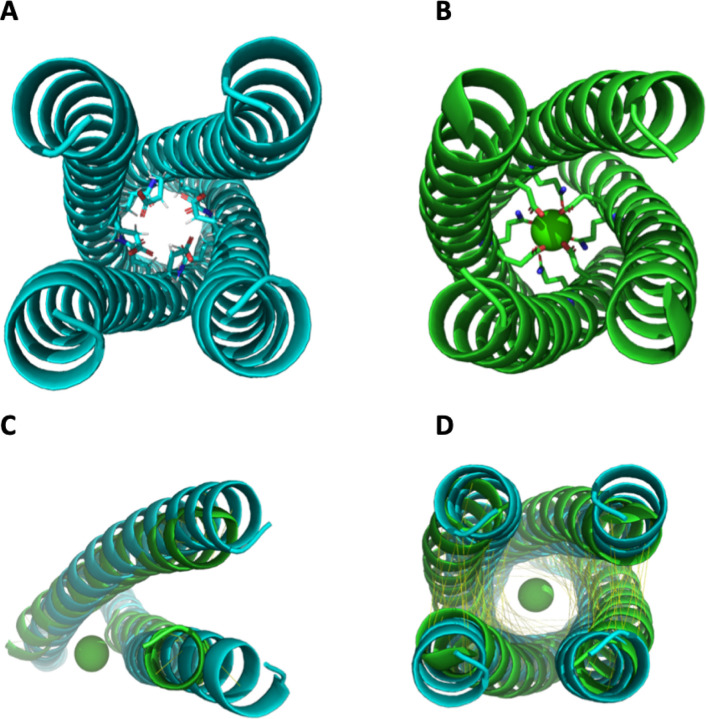
AlphaFold-Multimer model of the CCD tetramer shows similarity to experimental data. **A.** AlphaFold-Multimer model of the NSP4 CCD shown in cyan. Ca^2+^-interacting residues E120 and Q123 identified in **B.** the green experimental structure (PDB: 4WB4) are highlighted as licorice sticks. The Ca^2+^ ion present in the experimental structure is shown in green, but was not included in the AlphaFold-Multimer model. **C.** Alignment of split-states of tetrameric models resulted in an RMSD value of 3.844 Å. **D.** Alignment of both tetrameric models resulted in an RMSD value of 10.79 Å. Homologous atoms are connected by yellow lines. Renderings and analysis were completed using PyMOL.

**Figure 4:**
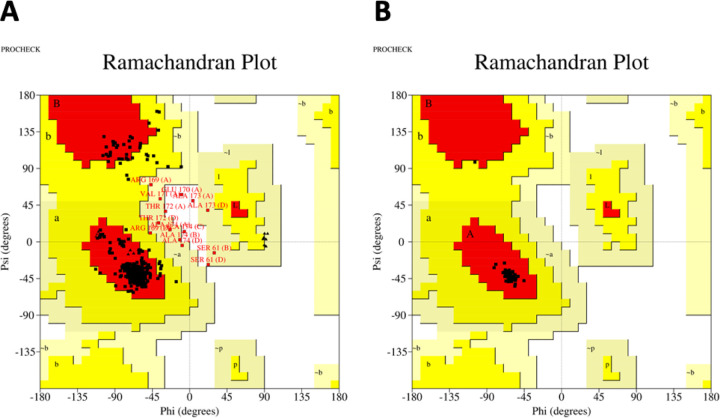
Alphafold-Multimer model of NSP4 CCD Tetramer model shows disallowed residues. **A.**Ramachandran plot of AlphaFold-Multimer model of WT SA11 NSP4 CCD tetramer produced by PROCHECK analysis. **B.** PROCHECK analysis of the experimental crystal structure of the CCD tetramer. Each dot corresponds to a single amino acid within the structure. Triangles represent glycine residues, and squares represent all other amino acids.

To properly compare our full-length tetrameric model with the established crystallographic tetramer model of the CCD of rotavirus SA11 NSP4, we excluded the VPD and residues 162–175 from the AlphaFold-Multimer model. Figures 3A and B reveal a discrepancy in the orientation of residues about the Ca^2+^ binding residues E120 and Q123. An initial alignment of the two models resulted in the high RMSD value of 10.79 Å. However, an alignment of their split-state resulted in an RMSD value of 3.844 Å, suggesting an increased degree of structural similarity between the two models. However, Ramachandran plots revealed a high degree of discrepancy between the torsional angles phi (φ) and psi (Ψ) of the backbone of the two models (Fig. 4). The AlphaFold-Multimer model had many residues consistent with beta-pleated sheet characteristics, motifs that are not associated with NSP4. Conversely, essentially all of the residues from the experimental structure of the tetramer have right-handed helical character, motifs that are heavily associated with NSP4. Furthermore, (Fig. 7A) the Ramachandran plot for the AlphaFold2 model showed that 0.6% of the residues were in structurally disallowed regions.

**Figure 5:**
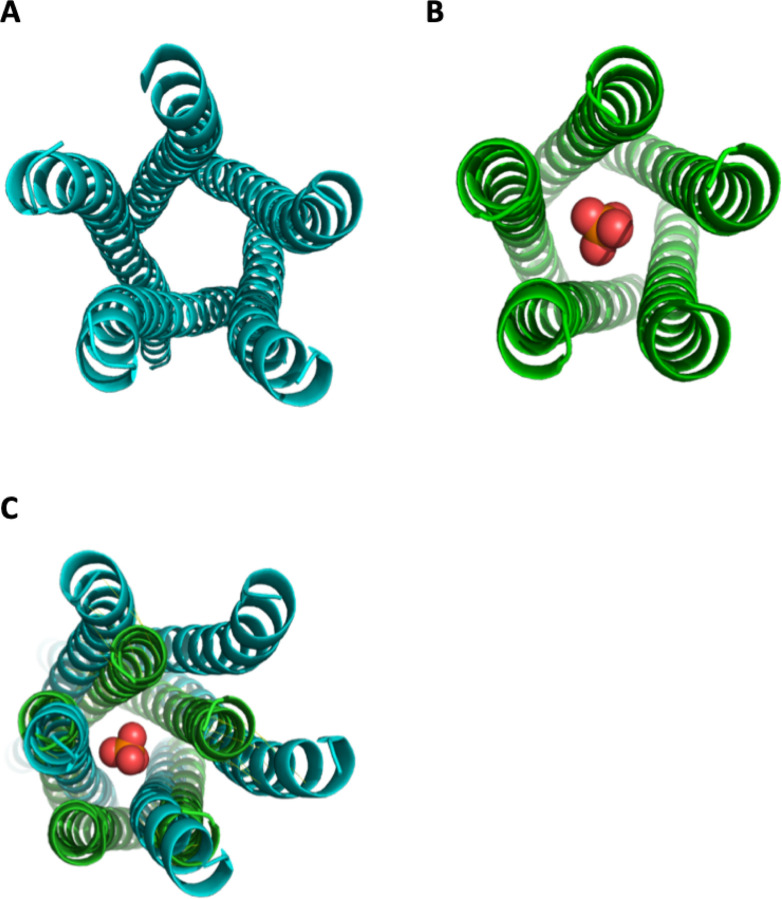
AlphaFold-Multimer model of the NSP4 CCD pentamer shows similarity to experimental data. **A.** AlphaFold-Multimer model of WT SA11 NSP4 CCD colored in cyan. **B.** Experimental model of the pentameric E120A/Q123A mutant of NSP4 (PDB:4WBA) shown in green with stabilizing buffer element, phosphate, rendered in space fill as red and orange. **C.** Alignment of the models resulted in an RMSD of 3.848 Å. Homologous atoms are connected by yellow lines. Renderings and analysis were completed using PyMOL.

Like the comparison of tetrameric models, we excluded the VPD and residues 162–175 from our full-length pentameric model, for proper comparison. Again, we observed some similarity at the amino-terminal end of the protein, and more divergence near the C-terminal CCD (Fig. 5). Aligning the two models resulted in an RMSD value of 3.848 Å, just 0.004 Å higher than that of the split-state tetramers. Panels A and B of Fig. 5 demonstrate the preserved rotational symmetry. Despite the structural similarities indicated by the acceptable RMSD value, the Ramachandran plots (Fig. 8) showed that the AlphaFold-Multimer model had many residues consistent with beta-pleated sheet characteristics, despite effectively all the residues from the pentameric crystal structure having right-handed helical character. Furthermore, Fig. 8A shows that 0.1 % of residues in the AlphaFold2 model were in structurally disallowed regions. Despite the overall increase in accuracy of these algorithms for membrane proteins, our data suggest that there is higher confidence for the predictions of the soluble portions of mixed-segment proteins than the membrane-associated sections.

**Figure 7:**
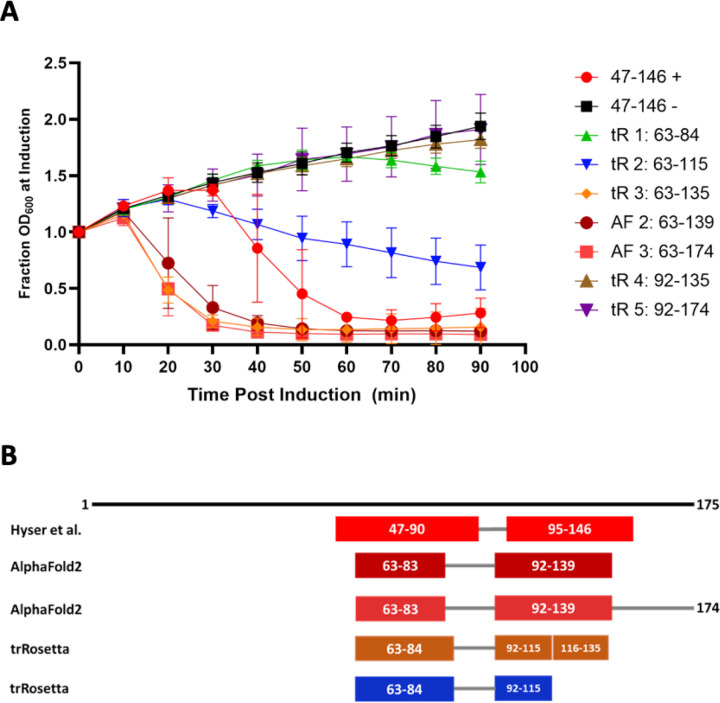
Selected model-based constructs show viroporin activity in an E coli surrogate assay. **A.** *E.coli* cells expressing truncation mutants of NSP4 of SA11 rotavirus predicted by either trRosetta (tR), or AlphaFold2 (AF), were assessed for viroporin function as described by (10). Data points represent the average of three independent experiments. Error bars represent standard deviation. **B.**Schematic of functional helices predicted from each computational method and their approximate position within the length of NSP4. Colors in the schematic correspond to Panel A. Predicted helices are represented as rectangles. Coils or disordered regions are represented as grey lines.

To determine if the segment demarcations of each of the NSP4 computational models could generate functional truncation mutants, as is seen with the current working model, we constructed versions of the SA11 NSP4 sequence informed by the segment limits of the trRosetta and AlphaFold2 models respectively [7, 10]. Truncation mutants were designed to include residues from the middle of the structure through the C-terminus, which have functionally been linked to the pathogenically-important virpororin activity of the protein [25–29]. Using our established E. coli surrogate system, each truncation mutant was co-expressed with lysozyme, and bacterial cultures monitored for lysis (Fig. 7) [10]. Compared to the construct from Hyser et. al, including residues 47 through 146, we observed similar viroporin activity from AlphaFold constructs including residues 63 to 139, and 63 to 174 respectively. The smaller of the two constructs exhibited 24 fewer amino acids that the comparable version from Hyser et al. From the trRosetta model, we observed function from constructs including residues 92 to 135, and 92–174, on par with what we saw from the AlphaFold versions. Interestingly, we noted an intermediate activity from a trRosetta construct including residues 63–115, but the function was not as robust as that seen for the other functional versions. Although it is not known whether these truncation mutants adopt a common topology in this bacterial assay, we were able to observe viroporin function from multiple NSP4 mutants based on their trRosetta and AlphaFold models.

**Figure 8:**
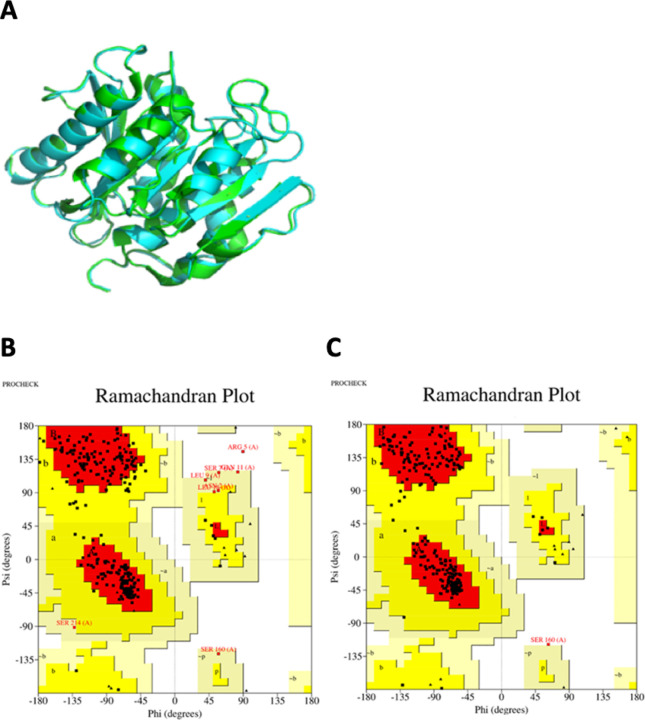
AlphaFold and experimental models of PETase show near congruency. **A.** AlphaFold (cyan) and experimental (green) models of polyethylene terephthalate degrading hydrolase (PETase (cyan) exhibiting an RMSD value of 0.304 Å (PDB: 6EQF). Homologous atoms are connected by yellow lines. **B.** Ramachandran plot of AlphaFold model of PETase produced by PROCHECK analysis. **C.** PROCHECK analysis of the experimental crystal structure of PETase. Each dot corresponds to a single amino acid within the structure. Triangles represent glycine residues, and squares represent every other type of amino acid.

**Figure 9:**
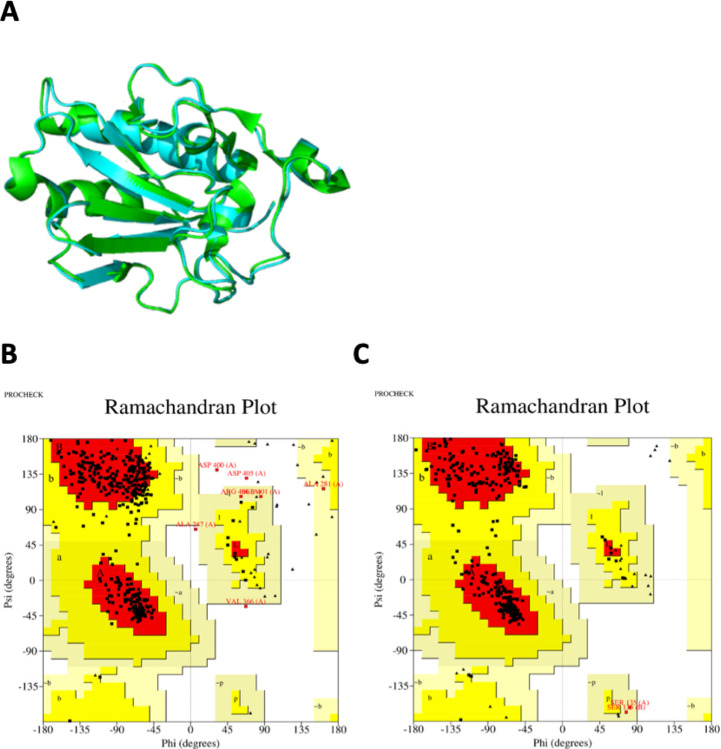
AlphaFold and experimental models of Human Sonic Hedgehog protein (SHH) show near congruency. **A.** AlphaFold (cyan) and experimental (green) models of SHH (cyan) exhibiting an RMSD value of 0.326 Å (PDB: 3M1N). Homologous atoms are connected by yellow lines. **B.** Ramachandran plot of AlphaFold model of SHH produced by PROCHECK analysis. **C.** PROCHECK analysis of the experimental crystal structure of PETase. Each dot corresponds to a single amino acid within the structure. Triangles represent glycine residues, and squares represent every other type of amino acid.

To illustrate AlphaFold2’s predictive capabilities from different organisms, we first generated models of the soluble PETase from the bacterium Ideonella sakaiensis (Fig. 8), and Human Sonic Hedgehog protein (SHH) (Fig. 9). We then compared the results with their corresponding experimental structures. The resulting RMSD values for PETase and SHH were 0.304 Å and 0.380 Å respectively. Though homologous amino acids were still connected by yellow lines, discrepancies between the predicted and experimental models were so minute that these lines were not easily visible. These data are further supported by Ramachandran plots of the models, showing similarity in both cases. Though the AlphaFold2 models have more residues in structurally disallowed regions, some stereochemical violations are to be expected in unrefined structure models.

**Figure 10:**
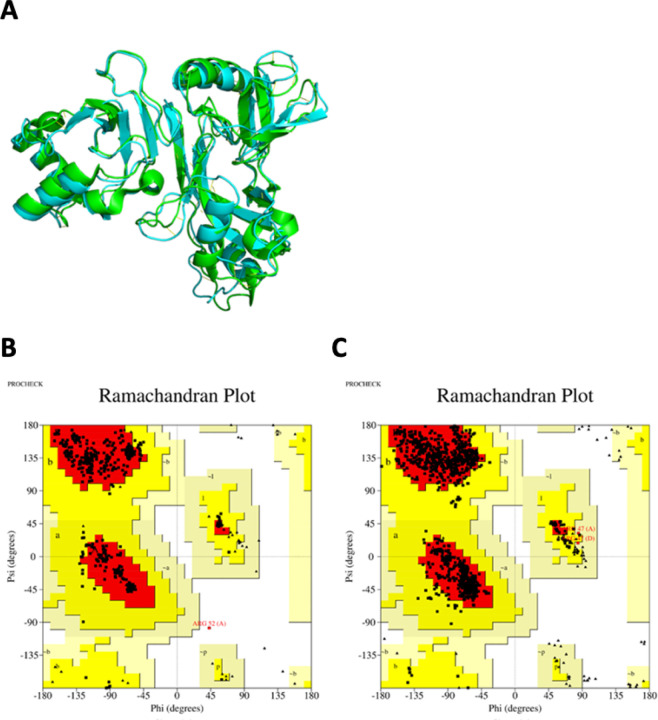
AlphaFold and experimental models of the N-terminal SARS-CoV-2 NSP2 show similarity. **A.** AlphaFold (cyan) and experimental (green) models of NSP2 (cyan) exhibiting an RMSD value of 1.617 Å (PDB: 7EXM). Homologous atoms are connected by yellow lines. **B.** Ramachandran plot of AlphaFold model of NSP2produced by PROCHECK analysis. **C.**PROCHECK analysis of the experimental crystal structure of NSP2. Each dot corresponds to a single amino acid within the structure. Triangles represent glycine residues, and squares represent every other type of amino acid.

Based upon the lack of virally-encoded proteins in the training data set of the AlphaFold program, we tested a soluble, viral protein from SARS-CoV2. Because the work by Heo et, al. has not yet been peer-reviewed, we compared their MD simulation-refined AlphaFold2 model (Fig. 10) of N-terminal SARS-CoV-2 NSP2 with the recently published crystal structure [9, 18]. An alignment of the two models resulted in an RMSD value of 1.617 Å. At this resolution, yellow lines connecting homologous molecules were difficult to see, indicating that the two structures were quite similar; these data are supported by their respective Ramachandran plots (Fig. 9A & B). Though 0.2% of residues in the AlphaFold2 model were in structurally disallowed regions compared to 0% of residues in the crystal structure, the AlphaFold2 model had 1.3% more residues in the most favored regions of the Ramachandran plot.

## Discussion

Machine learning based methods are a highly enticing alternative to slower, more expensive experimental ones. However, there remain some constraints to machine learning models, which rely on template-based modeling. Consequently, predictions largely focus on the average structure found within a library of homologous sequences, resulting in an inability to accurately capture differences in structure packing due to varying sidechains. Though both AlphaFold2 and trRosetta exclude viral proteins in their training datasets, the conservation of structural motifs in ion channels across the domains of life can still allow for meaningful modeling. We recognize that these algorithms are not intended for use on viral proteins, and that our models represent the currently capable limits of machine-learning based methods. Physics-based protein model refinement methods can address these shortcomings using conformational sampling around the predicted structures. Molecular dynamics (MD), a subset of protein model refinement, is one of the most successful methods for refining dubious structural features [16]. MD-refined AlphaFold2 structures allowed for accurate modeling of SARS-CoV-2 proteins early in the COVID-19 pandemic [17). Unfortunately, until methodology includes some variation of viral protein model refinement, NSP4 models cannot hold up to the established crystal structures.

Only limited insight can be drawn from modeling monomeric NSP4, as this protein is most functional as a tetramer or pentamer. It is likely that NSP4 only exhibits structural stability in these two oligomeric states, though versions of the protein containing both the VPD and CCD may adopt alternative oligomeric states. The direct comparison between the AlphaFold2 and trRosetta models sheds light on which currently available algorithm is better suited for modeling viroporins. AlphaFold2’s model predicted the CCD to be one long alpha helix, remarkably close to the previously proposed location of the CCD. The trRosetta model was unable to predict the CCD as a single motif, and instead split the entire protein into a series of helices. Though trRosetta is undoubtedly a cutting-edge tool in protein structure determination, these results speak to this algorithm’s relative inability to predict the structure of proteins underrepresented within its training dataset. The modest RMSD values, and problematic Ramachandran plots of the AlphaFold-Multimer models, reveal that there is much work to be done before machine-learning models alone can challenge crystallographic analysis for viral proteins. This is hardly surprising, given that predicting their structure is not AlphaFold’s intended purpose.

The advent of accurate and precise structure prediction algorithms marks a new era of proteomics. Here, we demonstrate a methodology in which anyone with a computer and stable internet connection can feasibly generate experimental-grade protein structures with little cost or time. We also showed that when used properly, structure prediction algorithms can potentially challenge experimentally obtained protein structures. Ongoing studies should attempt to refine our models of NSP4. This can first be done by utilizing the full version of AlphaFold; though the Colab notebook version of AlphaFold is user friendly, it is a simplified version of the full algorithm. Future work studies should resume our attempts to run the full version of AlphaFold via the Bates Leavitt HPCC. Our NSP4 models can also be improved by using protein model refinement, namely molecular dynamics simulations. Ongoing studies should also attempt additional other in vitro methods of structure determination, such as cryo-EM or NMR, due to their relative success in modeling membrane proteins. They may also re-attempt the more challenging task of crystallizing NSP4. Future studies should also explore generating models with RoseTTAFold, another highly accurate open-access algorithm featured at CASP14.

## Figures and Tables

**Figure 6:**
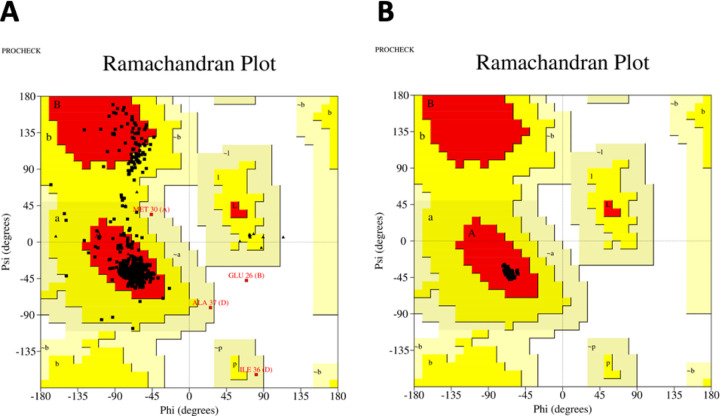
Alphafold-Multimer model of NSP4 CCD Pentamer model shows few disallowed residues. **A.**Ramachandran plot of AlphaFold-Multimer model of WT SA11 NSP4 CCD pentamer produced by PROCHECK analysis. **B.** PROCHECK analysis of the experimental crystal structure of the CCD pentamer. Each dot corresponds to a single amino acid within the structure. Triangles represent glycine residues, and squares represent all other amino acids.
